# Insight into the interaction between the RNA helicase CGH-1 and EDC-3 and its implications

**DOI:** 10.1038/s41598-021-99919-0

**Published:** 2021-10-13

**Authors:** Yong Zhang, Ke Wang, Kanglong Yang, Yunyu Shi, Jingjun Hong

**Affiliations:** 1 Hefei National Laboratory for Physical Sciences at the Microscale, School of Life Sciences, Division of Life Sciences and Medicine, University of Science and Technology of China, Hefei, 230027 Anhui People’s Republic of China; 2 Ministry of Education Key Laboratory for Membraneless Organelles and Cellular Dynamics, University of Science and Technology of China, Hefei, People’s Republic of China

**Keywords:** Biochemistry, Biological techniques

## Abstract

Previous studies indicated that the P-body components, CGH-1 and EDC-3 may play a crucial role in the regulation of lifespan in *Caenorhabditis elegans*. *Homo sapiens* DDX6 or *Saccharomyces cerevisiae* Dhh1p (CGH-1 in *C. elegans*) could form complexes with EDC3 (Edc3p in yeast), respectively, which is significant for translation inhibition and mRNA decay. However, it is currently unclear how CGH-1 can be recognized by EDC-3 in *C. elegans*. Here, we provided structural and biochemical insights into the interaction between CGH-1 and EDC-3. Combined with homology modeling, mutation, and ITC assays, we uncovered an interface between CGH-1 RecA2 domain and EDC-3 FDF-FEK. Additionally, GST-pulldown and co-localization experiments confirmed the interaction between CGH-1 and EDC-3 in vitro and in vivo. We also analyzed PATR-1-binding interface on CGH-1 RecA2 by ITC assays. Moreover, we unveiled the similarity and differences of the binding mode between EDC-3 and CAR-1 or PATR-1. Taken together, these findings provide insights into the recognition of DEAD-box protein CGH-1 by EDC-3 FDF-FEK motif, suggesting important functional implications.

## Introduction

Decapping of mRNA is a key step in eukaryotic cytoplasmic mRNA turnover/decay and therefore of gene expression. More common pathway for mRNA decay involves the removal of 5′ 7-methylguanosine (m7G) cap in the cytoplasm to allow for 5′-to-3′ exonucleolytic decay of mRNA. mRNAs associated with the decapping machinery can be assembled into mRNP granules termed as processing bodies (P-bodies). P-bodies are usually formed by a mechanism namely phase separation within eukaryotic cytoplasm. In addition to mRNA, P-bodies contain enzymes that are involved in mRNA turnover and play fundamental roles in mRNA decay (reviewed in^1,2^). DCP2, the catalytic subunit of the decapping enzyme, removes the 5′ cap structure from mRNA and inhibits translation and generally commits the mRNA to irreversible degradation, which is carried out by 5′-to-3′ exoribonuclease 1 (XRN1). The catalytic activity of DCP2 can be robustly stimulated by its essential coactivator DCP1. Other proteins such as DDX6, enhancer of decapping-3 (EDC-3), LSm14A, Pat, and the LSm1-7 complex so on, can modulate the recruitment and activity of the decapping complex (reviewed in^3,4^).

**Figure 1:**
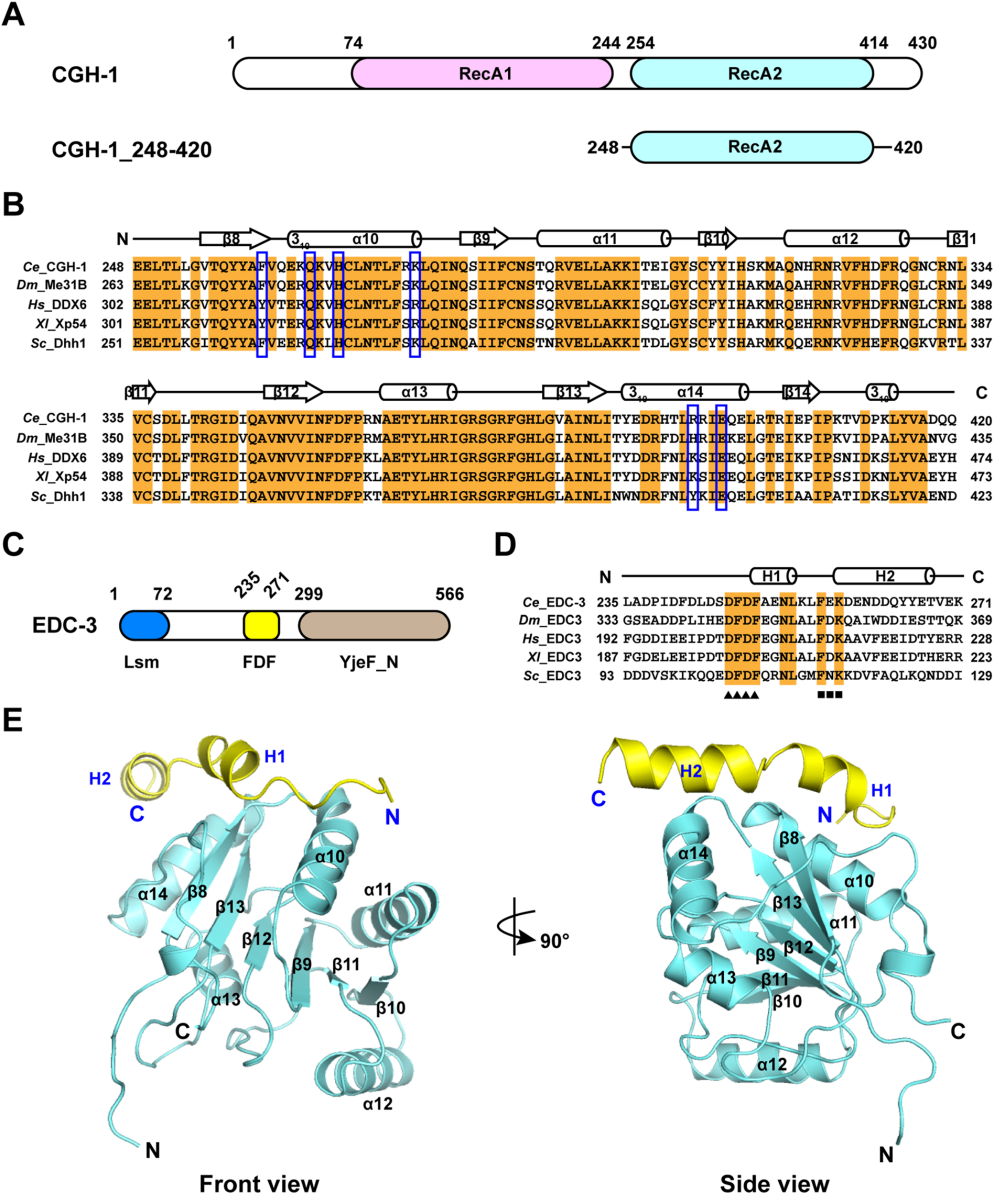
Structural model of CGH-1 RecA2 domain in complex with EDC-3 FDF-FEK. (**A**) Schematic view of domain architecture of *C. elegans* CGH-1. RecA1 and RecA2 domain are colored in violet and cyan, respectively. The construct of RecA2 domain employed in this work was also shown. (**B**) Sequence alignment of CGH-1_248–420_ and its orthologs. The residues for site-directed mutation in this study were marked with blue boxes. The secondary structures were also indicated in this panel. (**C**) Schematic view of domain architecture of *C. elegans* EDC-3. LSm domain, FDF domain, and YjeF-N domain are colored in blue, yellow and brown, respectively. (**D**) Sequence alignment of *Ce* EDC-3_235–271_ and its orthologs. DFDF and FxK are represented by triangles and squares, respectively. The second structures were also shown in this panel. (**E**) Overall view of this model shown in cartoon. CGH-1 RecA2 domain and EDC-3 FDF are colored in cyan and yellow, respectively.

The human DEAD (Asp-Glu-Ala-Asp) box DDX6 and its orthologs in *X. laevis* (Xp54), *D. melanogaster* (Me31B), *C. elegans* (CGH-1), and *S. cerevisiae* (Dhh1p) (Fig. 1A,B) play a critical role in posttranscriptional gene regulation by mediating both translational repression and mRNA decapping (reviewed in^5,6^). In *C. elegans*, CGH-1 could be detected throughout the life cycle, and it is a very important regulator of many life events, including miRNA mediated silencing, neuron development and mRNA turnover in P-bodies^7–9^.

In addition, EDC-3 (also called LSm16) is an enhancer of decapping and forms a network of interactions with the components of the mRNA decapping machinery. EDC-3 consists of an N-terminal LSm domain, a central FDF (Phe-Asp-Phe) domain, and a C-terminal YjeF-N domain (Fig. 1C,D)^10^. The LSm domain mediates DCP1 binding and P-body localization^11^, the FDF domain directly interacts with DEAD-box helicase Dhh1p in yeast and DDX6 in humans^12^. In humans, mRNA decapping may play an important role in neurodevelopment and in turn dysregulation of mRNA decapping is related to intellectual disability^13,14^. Functional analysis indicates that a homozygous variant in human EDC3 (EDC3^F54S^) that mechanistically fails to enhance DCP2 decapping activity is associated with autosomal recessive intellectual disability^13^. Moreover, bioinformatics analysis characterizes the role of EDC3 in mRNA decay and association of dysregulation of mRNA degradation with intellectual disability^14^. In *C. elegans*, however, the molecular and physiological function of EDC-3 has not been well understood, although it was reported that EDC-3 acts a pivotal part in modulating the aging and lifespan of *C. elegans*^15^.

Currently, we still don’t know in detail how EDC-3 recognizes CGH-1 in vitro and in *C. elegans*? A yeast two-hybrid analysis had previously found that CGH-1 and EDC-3 interact and that the LSm domain of EDC-3 is not required for this interaction^16^. Moreover, in *C. elegans*, a substantial increase was observed in the lifespan of *edc-3*(*ok1427*) mutants harboring a deletion in the *edc-3* locus. The corresponding mutant protein lacks 202 amino acids, including the conserved FDF domain of EDC-3^15^. This line of evidence implies that EDC-3 FDF mediated recruitment of CGH-1 might also be involved in the regulation of lifespan in *C. elegans*.

To dissect the structural and biological function relationship, the first step is to elucidate the interaction mechanism between CGH-1 and EDC-3 at the molecular or structural level. In this study, we aimed to investigate the interaction of EDC-3 with CGH-1 in vitro. Here, we expressed and purified recombinant CGH-1_248–420_ and purchased EDC-3 peptide from company as described in the section “Materials and methods” (Fig. 1A,C), measured their binding affinity by isothermal titration calorimetry (ITC) assay. We found that EDC-3_235–271_ binds to CGH-1_248–420_ with a dissociation equilibrium constant (*K*_D_) of approximate 0.34 μM. Based on homology modeling [we are not able to crystalize the complex of CGH-1_248–420_/EDC-3_235–271_], mutation and biochemical analyses, we demonstrated that EDC-3 FDF-FEK is anchored to CGH-1 RecA2 domain through a conserved hydrophobic surface. Additionally, GST-pulldown assays and in vivo colocalization experiment confirmed the physical interaction between CGH-1 and EDC-3. Intriguingly, the binding mode of EDC-3 with CGH-1 is different from that of CAR-1 or PATR-1 (Pat1 ortholog in *C. elegans*) with CGH-1. Altogether, these findings provide insights into the recognition of DEAD-box protein CGH-1 by EDC-3 FDF-FEK motif, suggesting valuable implications for the further research of mRNA decay or/and lifespan in *C. elegans*.

## Materials and methods

### Plasmids and constructs

Expression plasmids regarding CGH-1 and CAR-1 were constructed as previously described^17^. In brief, the cDNA fragments encoding CGH-1 RecA2 (residues 248–420), CAR-1 FDF-TFG (residues 184–268) were amplified by PCR from *C. elegans* genome library and cloned into the *Nde*I and *Xho*I site of a modified pET-28a (Novagen) vector (p28a), in which a thrombin protease cleavage site was removed. Mutants of CGH-1_248–420_ were individually generated through the MutanBEST kit (TaKaRa), and then confirmed by DNA sequencing.

The cDNA encoding a fragment of *C. elegans* EDC-3 (residues 230–566) plus a C-terminal His_6_ tag was synthesized by Sangon Biotech (Shanghai, China) Co., Ltd. The DNA fragment was then cloned into a pGEX-4T-1 expression vector using restriction enzymes *Bam*HI and *Xho*I, and finally confirmed by DNA sequencing.

### Protein expression and purification

CGH-1 protein expression and purification were performed described as in our most recent paper^17^. In brief, proteins were expressed in *E. coli* Gold (DE3) strain (Novagen). *E. coli* cultures with kanamycin were incubated at 37 °C till A_600_ reached about 1.0, and subsequently induced by 0.5 mM IPTG (isopropyl β-D-1-thiogalactopyranoside) at 16 °C overnight. Cells were resuspended in Buffer A (20 mM Tris–HCl, pH 7.5, 1 M NaCl), and then lysed using high pressure at 4 °C. Proteins were initially purified by Ni-chelating resin (Qiagen), and further purified by size exclusion chromatography (SEC) on a Superdex 75 column (GE healthcare) in Buffer A. The purified proteins were dialyzed into Buffer B (20 mM Tris–HCl, pH 7.5, 150 mM NaCl).

Both GST-tagged and His_6_-tagged fragment of EDC-3 protein was induced at 22 °C overnight in *E. coli* BL21 (DE3), initially purified by Ni-chelating resin (Qiagen) and further purified by SEC using a Hiload 16/60 Superdex 75 in a buffer containing 25 mM Tris–HCl, pH 7.5, and 500 mM NaCl, and followed by dialysis into phosphate buffered saline (PBS).

### Peptide preparation

A peptide containing the *C. elegans* EDC-3 FDF fragment (residues 235–271) and PATR-1 fragment (residues 30–67) were synthesized from GL Biochem (Shanghai, China) Co., Ltd, respectively. Stock solutions of peptide were prepared in Buffer B described above, and were adjusted to pH 7.5 ± 0.02 for subsequent experiments. The amino acid sequences of EDC-3 FDF peptide and PATR-1 peptide were shown as below:

- NH_2_-LADPIDFDLDSDFDFAENLKLFEKDENDDQYYETVEK-COOH (EDC-3 peptide);
- NH_2_-SDEDHNIFDDEFDAANDETFGGGLDNIGENAELENYAT-COOH (PATR-1 peptide).

### Homology modeling

The amino acid sequences of CGH-1 and EDC-3 were obtained from UniProt database (http://www.uniprot.org/), individually aligned with the orthologs using Clustal Omega^18^ and subsequently analyzed using ESPript 3.0^19^. The complex structure of *H. sapiens* DDX6 and EDC-3 (PDB code 2WAX) was employed as a template for homology modeling. Homology modeling was performed using the alignment and PDB file to run the script in Modeller program^20^. All structural figures were displayed using PyMOL (http://www.pymol.org/).

### Isothermal titration calorimetry

ITC assays were performed as previously described^17^. In brief, it was carried out on a MicroCal PEAQ-ITC (Malvern) at 20 °C with the following settings: reference power, 5 μcal/s; initial delay, 60 s; stir speed, 750 rpm; spacing time, 120 s. Proteins (or its mutants) and peptides were adjusted to 0.05 mM and 0.5 mM, respectively. Experiments were initialed by injection of 1 μl each of peptide into the sample cell (200 μl) filled with protein (or its mutants) solution, followed by 18 injections of 2 μl. The ITC data were fitted with a one-binding-site model using the Origin analysis software.

For the ITC experiment regarding *Ce*PATR-1 peptide, using the same buffer conditions and similar titration conditions as EDC-3 peptide. The thermodynamic parameters of titration results are summarized in Tables 1 and 2, respectively.

**Table 1:** Thermodynamic parameters of the CGH-1_248–420_ (or its mutants) bound to its ligand EDC-3_235–271_ by ITC assays.

Protein	Ligand	*K*_D_ (μM)	*N*	*ΔH* (kcal per mol)	*− TΔS* (kcal/mol)
CGH-1_248-420^WT^	EDC3_235-271^WT^	0.34 ± 0.02	1.07	*− *17.6 ± 0.09	8.94
CGH-1_248-420^F261A^	EDC3_235-271^WT^	1.16 ± 0.24	1.09	*− *14.1 ± 0.48	6.17
CGH-1_248-420^Q266A^	EDC3_235-271^WT^	1.75 ± 0.38	1.10	*− *12.1 ± 0.51	4.34
CGH-1_248-420^H269A^	EDC3_235-271^WT^	2.55 ± 0.32	1.03	*− *12.5 ± 0.34	4.97
CGH-1_248-420^K277A^	EDC3_235-271^WT^	2.74 ± 0.34	1.22	*− *15.2 ± 0.47	7.74
CGH-1_248-420^K277E^	EDC3_235-271^WT^	5.97 ± 0.51	1.12	*− *11.5 ± 0.30	4.48
CGH-1_248-420^R393A^	EDC3_235-271^WT^	2.07 ± 0.28	0.99	*− *17.3 ± 0.47	9.68
CGH-1_248-420^E396A^	EDC3_235-271^WT^	0.85 ± 0.11	1.00	*− *16.1 ± 0.31	7.92
CGH-1_248-420^4A^	EDC3_235-271^WT^	8.75 ± 3.64	0.79	*− *5.94 ± 0.89	*− *0.85
CGH-1_248-420^WT^	CAR-1_184-268^WT^	3.03 ± 0.21	0.89	*− *27.0 ± 0.43	19.6

**Table 2:** Thermodynamic parameters of the CGH-1_248–420_ (or its mutants) bound to its ligand *Ce*PATR-1_30–67_ by ITC assays.

Protein	Ligand	*K*_D_ (μM)	*N*	*ΔH* (kcal/mol)	*− TΔS* (kcal/mol)
CGH-1_248-420^WT^	PATR1_30-67^WT^	2.13 ± 0.38	0.88	*− *15.8 ± 0.62	8.16
CGH-1_248-420^F261A^	PATR1_30-67^WT^	6.62 ± 1.05	0.76	*− *13.0 ± 0.70	6.08
CGH-1_248-420^V262A^	PATR1_30-67^WT^	4.94 ± 0.79	0.84	*− *17.3 ± 0.82	10.1
CGH-1_248-420^Q266A^	PATR1_30-67^WT^	2.97 ± 0.29	0.86	*− *16.6 ± 0.41	9.17
CGH-1_248-420^H269A^	PATR1_30-67^WT^	2.67 ± 0.44	0.94	*− *15.4 ± 0.67	7.94
CGH-1_248-420^K277A^	PATR1_30-67^WT^	4.43 ± 0.42	1.07	*− *17.2 ± 0.57	10.0
CGH-1_248-420^K277E^	PATR1_30-67^WT^	8.29 ± 0.99	1.07	*− *19.6 ± 0.95	12.7
CGH-1_248-420^F355A^	PATR1_30-67^WT^	–	–	–	–
CGH-1_248-420^Y386A^	PATR1_30-67^WT^	4.19 ± 0.58	0.85	*− *14.2 ± 0.56	6.95
CGH-1_248-420^R393A^	PATR1_30-67^WT^	4.22 ± 0.62	0.80	*− *16.6 ± 0.69	9.42
CGH-1_248-420^E396A^	PATR1_30-67^WT^	2.96 ± 0.34	0.93	*− *16.3 ± 0.47	8.89
CGH-1_248-420^4A^	PATR1_30-67^WT^	2.06 ± 0.27	0.79	*− *15.4 ± 0.44	7.76

### GST-pulldown assays

To test if GST-tagged EDC-3 can pulldown CGH-1 protein, about 100 μg protein, GST alone or GST-EDC-3_230–566_, was incubated with GST resin (GE Healthcare) in PBS for 1 h. The resin was then washed three times with the same buffer. Subsequently, the resin was incubated with ~ 50 μg recombinant CGH-1_248–420_ protein or its 4A mutant in the same buffer for 2 h. The resin was then washed five times using the same buffer. The captured proteins in resin were finally heated and analyzed using 12% SDS-PAGE.

To investigate whether CAR-1_184–268_ can affect CGH-1_248–420_ binding to EDC-3_230–566_, approximate 100 μg GST-tagged EDC-3_230–566_ was incubated with GST resin, followed by three times of wash in PBS. The resin was then incubated with 50 μg CGH-1_248–420_ in the absence or presence of the same amount of CAR-1_184–268_ for 2 h, followed by five times of PBS wash, and captured proteins were finally analyzed as described above.

To investigate whether *C. elegans* PATR-1 peptide will affect the CGH-1 binding to GST-tagged EDC-3_230–566_ protein, approximate 100 μg GST-tagged EDC-3_230–566_ was incubated with GST resin, followed by three times of wash in PBS. The resin was then incubated with 50 μg CGH-1_248–420_ without PATR-1 peptide (0 μM) or with varied concentration of PATR-1 peptide (12.5, 25, or 50 μM) for 2 h, and was washed five times in PBS. The captured proteins in resin were finally heated and analyzed using 12% SDS-PAGE.

### *C. elegans* strains

Bristol Strain N2 was used as the standard wild type strain. All strains were incubated on nematode growth medium (NGM) plates seeded with OP50 at 20 °C. (SHG1686) CGH-1(*ustIS217 III*[*gfp::cgh-1*]), (SHG1687) EDC-3(*ustIS218 I*[*3xflag::mCherry::edc-3*]), (SHG1689) CGH-1(*ustIS217 III*[*gfp::cgh-1*]); EDC-3(*ustIS218 I*[*3xflag::mCherry::edc-3*]).

### Construction of sgRNA expression plasmids

We manually searched for target sequences consisting of G(N)19NGG near start codon of genes. To construct sgRNA expression vector, the 20 bp *unc-119* sgRNA guide sequence in the *pU6::unc-119* sgRNA(F + E) vector was replaced with different sgRNA guide sequences. Primer sequences are listed in Supplementary Table S1.

### Construction of donor plasmids

For in situ transgene expressing 3xFLAG::mCherry::EDC-3 or GFP::CGH-1 plasmid, a 1.5 kb left arm and 1.5 kb right arm was PCR amplified from N2 genomic DNA. ClonExpress^®^ MultiS One Step Cloning Kit (Vazyme C113-02, Nanjing, China) was used to connect these fragments.

### Microinjection

Plasmid mixtures containing 50 ng/μl sgRNA#1, 50 ng/µl sgRNA #2, 50 ng/µl sgRNA #3, 50 ng/µl Cas9 II expressing plasmid, 50 ng/µl donor plasmid, and 5 ng/µl pCFJ90 were co-injected into N2 animals. Plasmid mixtures were microinjected into the gonads of late young adult *C. elegans*. After recovering from injection, each worm was placed onto an individual OP50 plate.

### Screening for transgene by PCR

Three days after injection, F1 animals expressing GFP marker were transferred to individual NGM plates and allowed to produce progeny for 2 or 3 days. Progeny of F1 were collected with 50 ml DNA lysis buffer (500 µg/ml Proteinase K, 100 mM NaCl, 50 mM Tris, 20 mM EDTA, and 1% SDS), and screened by PCR amplification with designed primers. Primer sequences are listed in Supplementary Table S1.

### Imaging

Images were collected using Leica DM4B and M165 FC microscopes.

## Results

### CGH-1 RecA2 interacts with EDC-3 FDF-FEK domain in vitro

Previous studies in *H. sapiens* and *S. cerevisiae* revealed that DEAD-box RNA helicases DDX6 and Dhh1p mediate protein–protein interactions, depending on the recruitment of specific interacting partners (reviewed in^21^). CGH-1 is similar to its orthologs DDX6/Dhh1p, which contains two RecA-like domains connected by a short liker (Fig. 1A). In our most recent study, we have demonstrated in vitro that the ATPase activity of CGH-1 can be robustly stimulated by the MIF4G domain of NTL-1a (Not1 in yeast and CNOT1 in humans) in the presence of poly(U) RNA and ATP^17^. The Ccr4-Not deadenylase complex is responsible for the main ploy(A) removal activity in *C. elegans*^22^. We have also investigated in vitro that *C. elegans* CGH-1/CAR-1 (Scd6p in yeast and LSm14A in humans) interface^17^.

**Figure 2:**
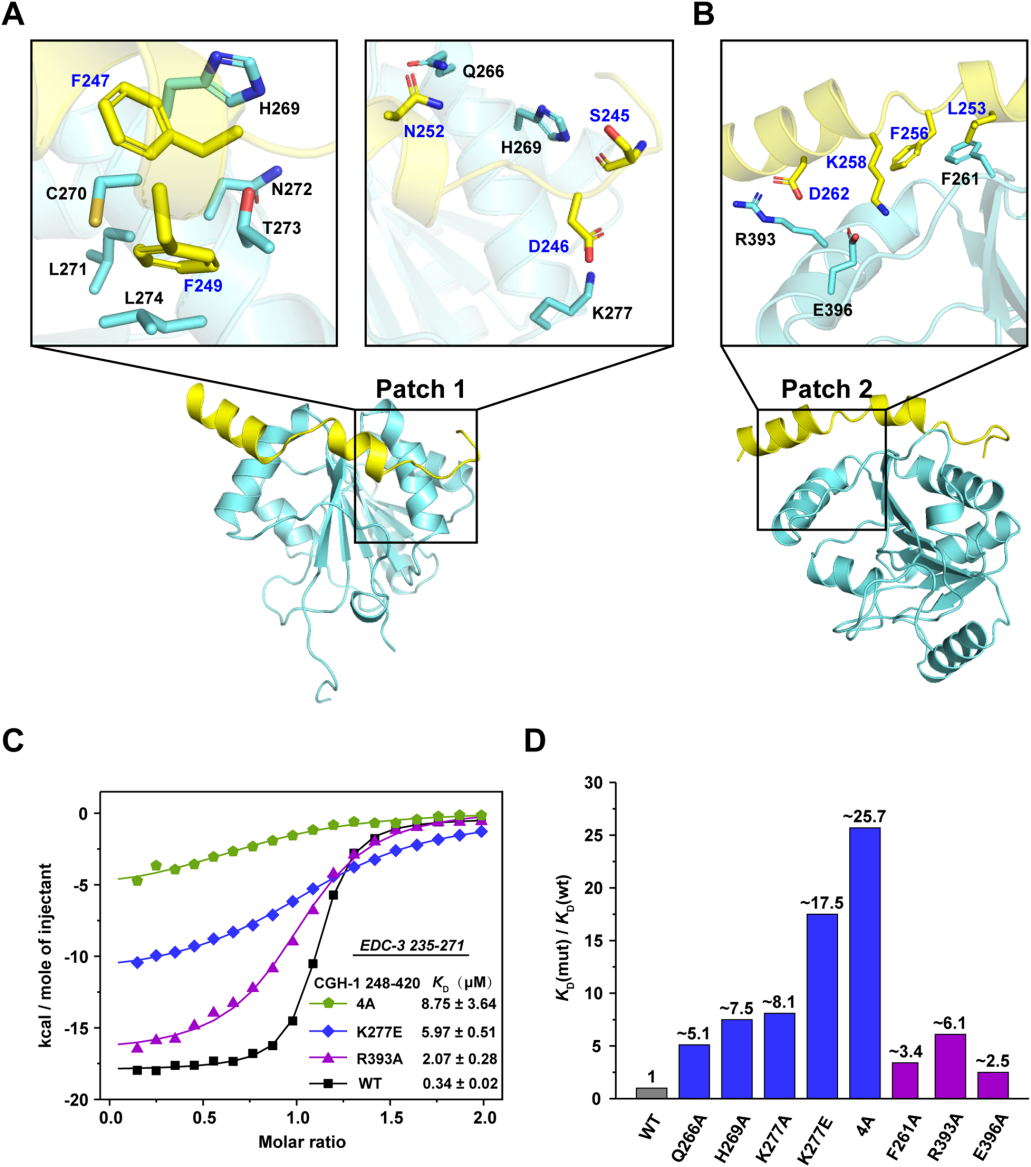
The interface of CGH-1 (cyan) and EDC-3 (yellow). (**A**) Interaction details of the Patch 1. Amino acids are shown as side chains. (**B**) Interaction details of the Patch 2. (**C**) Representative curves of ITC assays. (**D**) Effects for the mutations on the binding affinity between CGH-1 RecA2 domain and EDC-3 FDF-FEK peptide.

**Figure 3:**
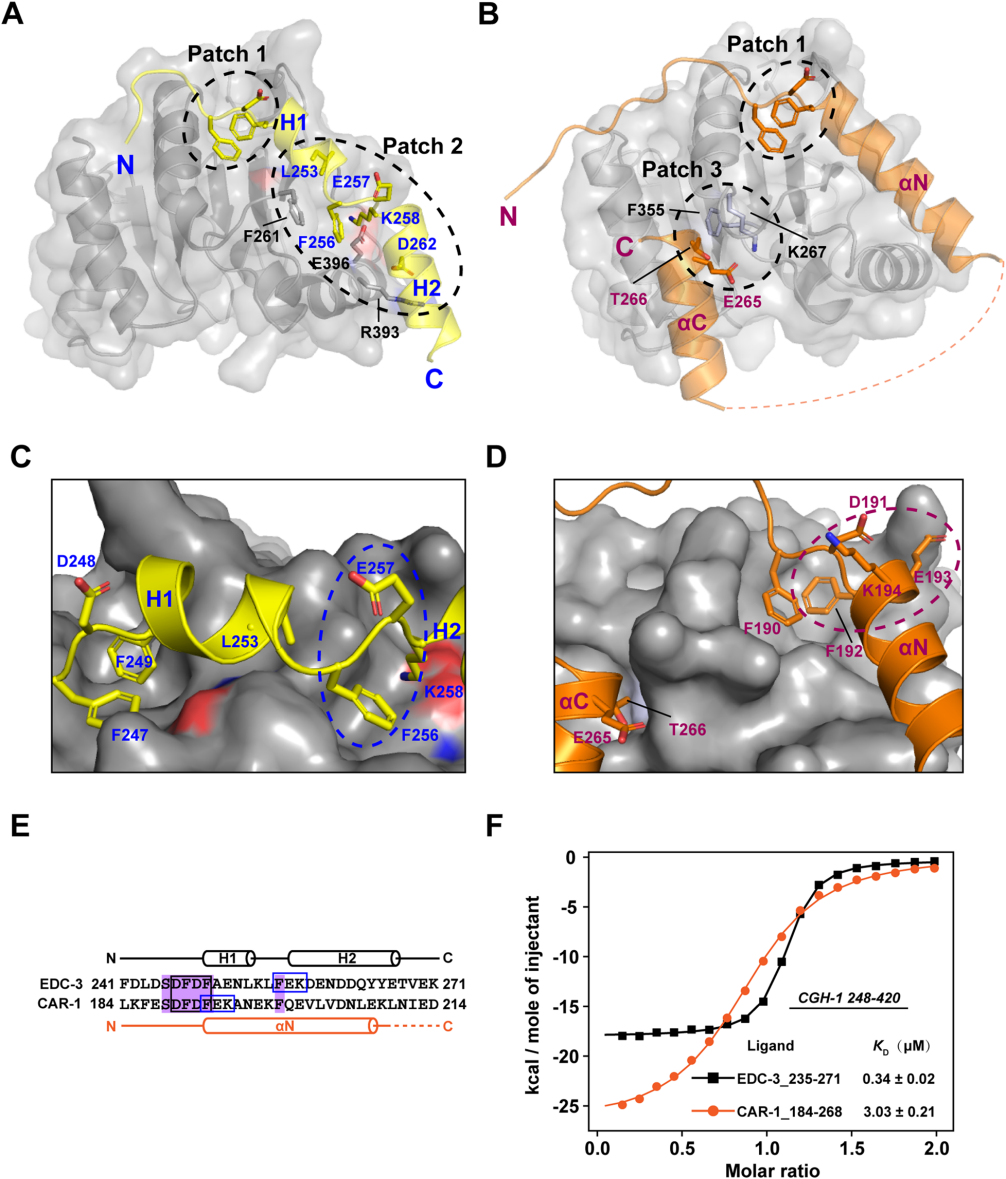
Similarity and differences of the binding mode of EDC-3 and CAR-1 for CGH-1. (**A**) The binding mode between CGH-1 RecA2 domain and EDC-3 FDF. Amino acids in the Patch 2 are represented by the side chains. (**B**) The binding mode between CGH-1 RecA2 domain and CAR-1 FDF-TFG. Amino acids in the Patch 3 are represented by the side chains. (**C**) EDC-3 FDF and FEK binding pocket. CGH-1 RecA2 domain is presented as a surface. FEK is marked with blue dotted ellipse. (**D**) CAR-1 FDF and TFG binding pocket. (**E**) Sequence alignment of EDC-3_241–271_ and CAR-1_184–214_. The conserved residues SDFDF is marked with a pink box, and FEK is marked with a blue box. Second structures were shown on the top. (**F**) ITC curves: titrating EDC-3_235–271_ and CAR-1_184–268_ to CGH-1_248–420_, respectively. The ITC data for EDC-3 in Figs. 2C and 3F are the same data.

**Figure 4:**
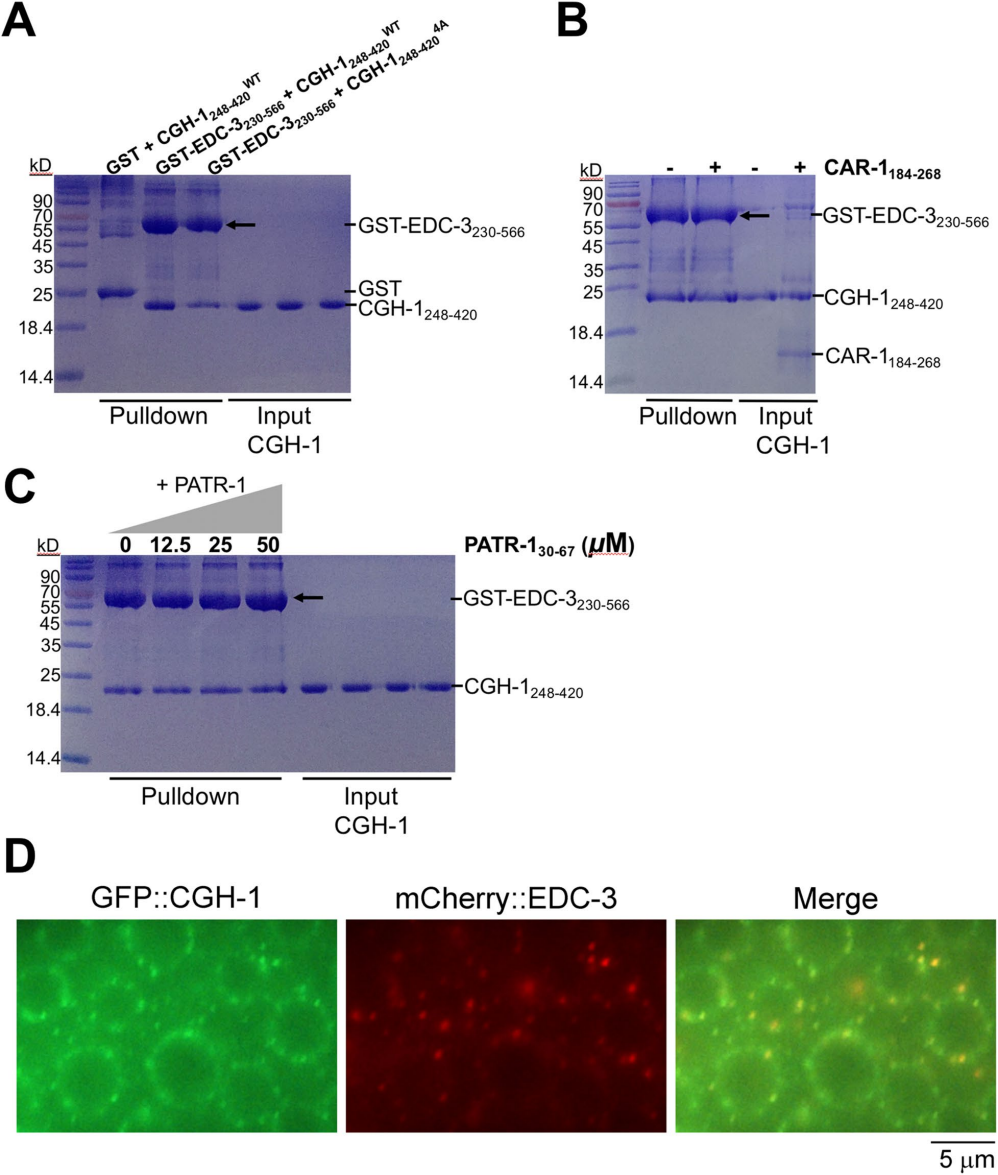
In vitro and in vivo interaction between *C. elegans* CGH-1 and EDC-3. (**A**) GST-pulldown assays of in vitro interaction between GST-EDC-3_230–566_ and CGH-1_248–420_ (WT or 4A mutant). For clarity, only the input of CGH-1 proteins was shown in SDS-PAGE analysis. (**B**) GST-pulldown assays of GST-EDC-3_230–566_ and CGH-1_248–420_ (WT) without or with co-incubation of recombinant CAR-1_184–268_ protein. (**C**) GST-pulldown assays of GST-EDC-3_230–566_ and CGH-1_248–420_ (WT) without or with co-incubation of increased concentration (12.5, 25, 50 μM) of PATR-1_30–67_ peptide. Note: PATR-1 peptide is difficult to be seen in this SDS-PAGE analysis because of its small molecular weight. (**D**) Subcellular co-localization of GFP::CGH-1 and 3xFLAG::mCherry::EDC-3 in *C. elegans*. In adult germ cells, GFP::CGH-1 and 3xFLAG::mCherry::EDC-3 were concentrated in foci that were distributed in a perinuclear pattern around nuclei.

In addition to NTL-1a and CAR-1 and their orthologs, the complex formed by DDX6/Dhh1p and EDC-3 is vital for the translational repression, mRNA decay, and P-body assembly and localization^23–25^. In one recent study, it has been implicated that CGH-1 and EDC-3 might be involved in the regulation of lifespan during aging in *C. elegans*^15^. However, how EDC-3 interacts with CGH-1 in detail is unclear. Based on the studies of EDC-3 orthologs, we assumed that CGH-1 could also bind to EDC-3 FDF motif via its RecA2 domain. To this end, we expressed and purified the recombinant CGH-1_248–420_ containing the RecA2 domain (Fig. 1A). As shown by the results of SEC and SDS-PAGE analysis, the quality for proteins is pure and uniform (Supplementary Fig. S1). To confirm this hypothesis, we next tested the EDC-3 FDF peptide for its ability to bind to CGH-1_248–420_ by performing the isothermal titration calorimetry (ITC) assays. Remarkably, the results showed that the EDC-3 FDF peptide directly binds to CGH-1_248–420_ with a dissociation equilibrium constant (*K*_D_) of ~ 0.34 μM and an *N* value of ~ 1 (Figs. 2C and 3F, and Table 1). Additionally, a GST-tagged fragment, which includes the central FDF and C-terminal YjeF-N domain of EDC-3 (EDC-3_230–566_), but not GST alone, can interact with CGH-1_248–420_ in a GST-pulldown assay (Fig. 4A). Overall, we conclude that CGH-1 RecA2 domain interacts with EDC-3 FDF domain in vitro.

### A model of CGH-1 RecA2 in complex with EDC-3 FDF-FEK

To elucidate the recognition mechanism of CGH-1 RecA2 domain by EDC-3 FDF, we tried to co-crystallize CGH-1_248–420_ with EDC-3 FDF peptide, but didn’t result in co-crystals. We then performed homology modeling by the Modeller program^20^. The model was produced using the complex crystal structure of human DDX6 and EDC3 (PDB code 2WAX) as the template due to the high sequence similarity (Fig. 1B,D,E).

We superimposed the completed model to the crystal structure of apo CGH-1 RecA2 domain, which has been recently determined by us (PDB code 7DTJ^17^), giving a root-mean-square-deviation (RMSD) of ~ 0.75 Å over the backbone Cα atoms, suggesting that the resulting model is well consistent with our determined crystal structure of CGH-1 RecA2. In the model, CGH-1 RecA2 domain consists of the typical RecA-like folds in topology similar to other DEAD-box RNA helicases (Fig. 1E). EDC-3 FDF is embedded into the shallow groove formed on the helices α10 and α14 of CGH-1 RecA2 domain, and folded into the helices H1 and H2 (Fig. 1E).

### The interface between CGH-1 RecA2 and EDC-3 FDF fragment

In our model, there are two continuous binding sites (Patch 1 and Patch 2) between CGH-1 RecA2 and EDC-3 FDF fragment (Figs. 2A,B, 3A). For Patch1, the phenylalanine Phe_247_ and Phe_249_ of the EDC-3 FDF (Phe_247_, Asp_248_, and Phe_249_) motif occupy the hydrophobic pocket (composed of His_269_, Cys_270_, Leu_271_, Asn_272_, Thr_273_, and Leu_274_) of the CGH-1 RecA2 domain (Fig. 2A). In addition, the residues Ser_245_, Asp_246_, and Asn_252_ of EDC-3 may interact with His_269_, Lys_277_, and Gln_266_ of CGH-1 by hydrogen bond or electrostatic interactions, respectively (Fig. 2A). For Patch 2, the residues Leu_253_ and Phe_256_ of EDC-3 may mediate the hydrophobic interactions with Phe_261_ of CGH-1 (Fig. 2B). In addition, the residues Lys_258_ and Asp_262_ of EDC-3 may mediate the electrostatic interactions with Glu_396_ and Arg_393_ of CGH-1, respectively (Fig. 2B).

To further confirm our structural model, we performed ITC experiment to determine the binding affinity of EDC-3 FDF peptide for CGH-1_248–420_ WT or its mutants. The substitution of each of CGH-1 residues such as Phe_261_, Gln_266_, His_269_, with Ala weakened the binding affinity by a factor of ~ 3.4–7.5 (Fig. 2C,D, Table 1, and Supplementary Fig. S2). Particularly, the substitutions of His_269_, Cys_270_, Thr_273_, and Leu_274_ to Ala (hereafter designated as 4A) in the hydrophobic pocket of CGH-1 severely weakened the binding affinity by a factor of ~ 25.7 (Fig. 2C,D, Table 1, and Supplementary Fig. S2), indicating that the 4A mutation of CGH-1 dramatically disrupted the interaction between CGH-1 and EDC-3 peptide. In line with data, less amount of the 4A mutant of CGH-1_248–420_ binds to GST-tagged EDC-3_230–566_ than wild-type CGH-1_248–420_ does in a GST-pulldown assay (Fig. 4A). These results clearly suggested that the hydrophobic interactions mediated by CGH-1 hydrophobic pocket and EDC-3 FDF motif play a very important role in the recognition of CGH-1 by EDC-3 (Fig. 2A).

In addition, the substitution of Lys_277_ with Ala (K277A) or Glu (K277E) in CGH-1 weakened the binding affinity by factors of ~ 8.1 and ~ 17.5, respectively (Fig. 2C,D, Table 1, and Supplementary Fig. S2), supporting our structural model that Lys_277_ of CGH-1 mainly mediates the electrostatic interaction with Asp_246_ of EDC-3 for stabilization between CGH-1 and EDC-3 (Fig. 2A). Moreover, in line with our model that Arg_393_ of CGH-1 may electrostatically interact with Asp_262_ of EDC-3, the substitution of Arg_393_ with Ala (R393A) decreased the binding affinity by a factor of ~ 6.1 (Fig. 2B–D). The mutation of Glu_396_ to Ala (E396A) also weakened the binding affinity, by a factor of ~ 2.5 (Fig. 2B,D). Taken together, these ITC titration results are well consistent with the interfaces provided by the model, indicating that EDC-3 FDF-FEK is anchored to CGH-1 in a specific manner.

### Co-localization of CGH-1 and EDC-3 in *C. elegans*

A previous yeast two-hybrid analysis indicated that CGH-1 and EDC-3 interact and that the LSm domain of EDC-3 is not required for this interaction^16^. In line with this result, in vitro pulldown assays also found that the EDC-3_230–566_ fragment excluding the LSm domain binds to CGH-1 RecA2 domain (Fig. 4A). Moreover, current models based on in vitro ITC assays suggest that EDC-3 should have a high affinity for CGH-1 in vivo.

To investigate the interaction of CGH-1 and EDC-3 in *C. elegans*, GFP::CGH-1 and 3xFLAG::mCherry::EDC-3 in situ were constructed via CRISPR/Cas9 technology^26^. In adult germ cells, GFP::CGH-1 and 3xFLAG::mCherry::EDC-3 were concentrated in foci which were distributed in a perinuclear pattern around nuclei. We crossed GFP::CGH-1 with 3xFLAG::mCherry::EDC-3 and found that a large portion of CGH-1 co-localized with EDC-3, supporting a physical interaction between the two proteins in vivo (Fig. 4D).

### The interaction between *C. elegans* PATR-1 and CGH-1

Structural analysis of the yeast Pat1–Dhh1 complex reveals how Pat1 recognizes Dhh1 via its N-terminal Phe-Asp-Phe (FDF) motif^23^. Sequence alignment indicates that the yeast Pat1 DFDF motif is not conserved, and the corresponding residues are DDDW (*H. sapiens* and *X. laevis*), NGDW (*D. melanogaster*), and NAEL (*C. elegans*), respectively (Supplementary Fig. S3A). It has been previously demonstrated by Co-immunoprecipitation assays combined with mutation that the hydrophobic Tryptophan (Trp_46_) in the Asp-Trp (DW) motif of human PATL1 (Hs Pat1b) is target to the FDF-binding site on DDX6^23^. A substitution of Trp_46_ to Ala (W46A) or Asp (W46D) in the YFP-tagged PATL1 impaired the interaction with human HA-tagged DDX6^23^.

In *C. elegans*, Boag et al.^8^ demonstrated by cell biology that CGH-1 associates with PATR-1 in *patr-1*-dependent somatic P bodies. In contrast, *patr-1* is not required for the formation of storage bodies in developing oocytes^8^. During oogenesis, CGH-1 associates primarily with CAR-1 and other regulators, and with and protects particular translationally regulated maternal mRNAs to form functional storage bodies or P granules^8,27^.

**Figure 5:**
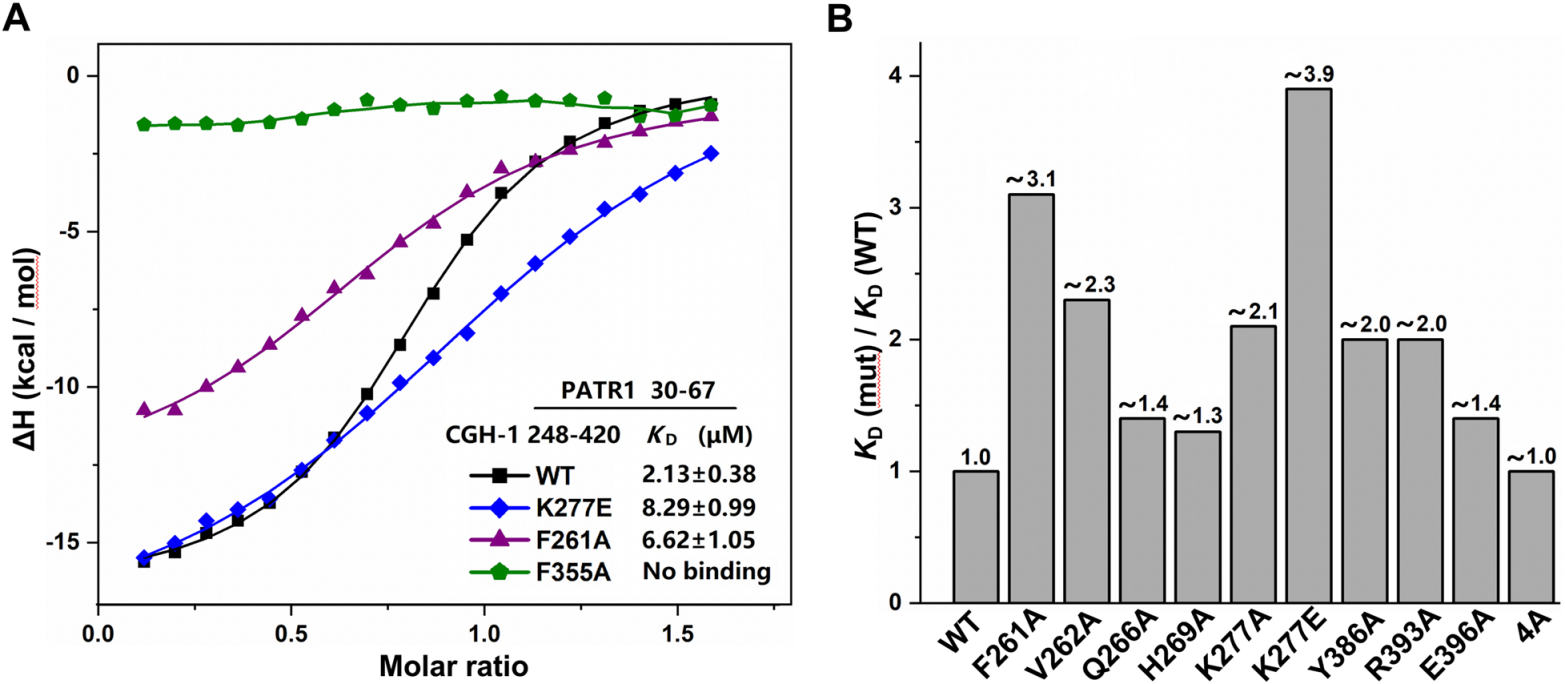
In vitro interaction between CGH-1 and *Ce*PATR-1_30–67_ peptide. (**A**) Representative curves of ITC assays. (**B**) Effects for the CGH-1 mutations on the binding affinity between CGH-1 RecA2 domain and *Ce*PATR-1 peptide.

To investigate in vitro interaction between *Ce*PATR-1 and CGH-1, we performed ITC assays to measure the binding affinity using a synthetic PATR-1 peptide (*Ce*PATR-1_30–67_) and recombinant fragment of CGH-1 (CGH-1_248–420_). The results indicated that the *Ce*PATR-1_30–67_ peptide directly binds to wild-type CGH-1_248–420_ with a *K*_D_ of approximate 2.1 μM (Table 2, Fig. 5A, Supplementary Fig. S4). To further identify the possible PATR-1-binding interface of CGH-1, we also measured the binding affinity between *Ce*PATR-1 peptide and CGH-1 mutants. The ITC data are summarized in Table 2, Fig. 5, and Supplementary Fig. S4.

In the structural model of CGH-1 and EDC-3, the two phenylalanine residues (Phe_247_ and Phe_249_) of EDC-3 reside the hydrophobic patch which consists of His_269_, Cys_270_, Thr_273_ and Leu_274_ of CGH-1 (Fig. 2A). In line with the structural model, the ITC data indicated that the 4A mutant dramatically impaired the binding affinity between EDC-3 and CGH-1 (Fig. 2C,D). Since the DFDF motif is absent in the amino acid sequence of *C. elegans* PATR-1, we hypothesize that the hydrophobic patch which consists of four CGH-1 residues His_269_, Cys_270_, Thr_273_ and Leu_274_ is not important for its interaction with PATR-1. To test this hypothesis, we prepared the four-alanine mutant (4A) and performed ITC experiment. To the end, we demonstrated that the 4A mutant almost has the same binding affinity with the wildtype CGH-1 protein (Table 2, Fig. 5B, Supplementary Fig. S4). In other words, the four residues (His_269_, Cys_270_, Thr_273_ and Leu_274_) are not involved in the interaction with *Ce*PATR-1_30–67_ peptide. Additionally, the mutation of each CGH-1 residue Gln_266_, His_269,_ or Glu_396_ to Ala showed slight decrease in the binding affinity when compared to that of wildtype (Table 2, Fig. 5B, Supplementary Fig. S4).

Instead, the substitution of each CGH-1 residue of Phe_261_, Val_262_, and Tyr_386_ to alanine, reduced the binding affinity by factors of 2–3, indicating that Phe_261_, Val_262_ and Tyr_386_ may be involved in the hydrophobic interaction with *Ce*PATR-1. Additionally, the mutation of Lys_277_ to Ala (K277A) or Glu (K277E) decreased the binding affinity by factors of approximate 2.1 and 3.9, respectively. Moreover, substitution of Arg_393_ to alanine (R393A) weakened the binding affinity by a factor of 2.0. These results indicated that Lys_277_ and Arg_393_ may mediate electrostatic interaction with *Ce*PATR-1 peptide. Similarly, Lys_277_ and Arg_393_ have also been found important to interact with EDC-3 (this work) and CAR-1^17^.

We recently identified a highly conserved FDF (Phe-Asp-Phe) motif between beta-strand 12 and alpha-helix 13 in CGH-1 RecA2 domain but the function is currently unclear (Fig. 1B)^17^. Mechanistically, Phe_355_ in CGH-1 may mediate hydrophobic interaction with Thr_266_ in the TFG motif of CAR-1 and a mutation of Phe_355_ to Ala (F355A) in CGH-1 decreased the binding affinity by a factor of approximate 5 as measured by ITC assay^17^. Since PATR-1N-terminus has also a TFG motif, we hypothesize that PATR-1 may also interact with CGH-1 via similar binding mechanism (Supplementary Fig. S3B and C). Strikingly, the F355A mutant of CGH-1 shows undetectable binding to PATR-1 peptide in our ITC assay (Fig. 5A, Supplementary Fig. S4). These data indicate that Phe_355_ play a very important role in the recognition of PATR-1 by CGH-1, and the F355A mutation in the FDF motif of CGH-1 almost abolishes the binding of *Ce*PATR-1_30–67_ peptide to CGH-1_248–420_ in vitro assay. Taken together, these results imply that PATR-1 TFG-binding site is similar to CAR-1 TFG-binding site on the CGH-1 RecA2 domain.

Finally, we ask whether CAR-1 or PATR-1 peptide can affect the binding of CGH-1 to EDC-3? Co-incubation of the same amount of CGH-1_248–420_ and CAR-1_184–268_ with GST-EDC-3_230–566_ showed little or no effect in the binding of CGH-1_248–420_ to EDC-3_230–566_ in a GST-pulldown assay (Fig. 4B). Moreover, co-incubation of increased amount of *Ce*PATR-1_30–67_ peptide with the solution of CGH-1_248–420_ and GST-EDC-3_230–566_ does not impair the interaction of CGH-1 with GST-tagged EDC-3_230–566_ in a GST-pulldown assay (Fig. 4C). These data may reflect a fact that EDC-3 has a higher affinity than CAR-1 or PATR-1 when they are binding to CGH-1 in vitro, as confirmed by our ITC assays.

## Discussion

### The similarities and differences of the binding mode between EDC-3 and CAR-1 for CGH-1

In *C. elegans*, CAR-1 is a germline specific cytokinesis, apoptosis, RNA-binding protein^27,28^, and contains three conserved domains: N-terminal Sm-like (Lsm) domain, central domain with FDF, FFD, TFG motifs, and C-terminal RGG box^28^. Our most recent works have delineated the recognition mechanism of CGH-1 by CAR-1^17^. By ITC assays, we found that the binding affinity of EDC-3_235–271_ is approximately ninefold stronger than that of CAR-1_184–268_ when they are bound to CGH-1_248–420_ (indicated by *K*_D_, 0.34 μM vs 3.03 μM; Fig. 3F) in the same buffer conditions. It has also been demonstrated that a CAR-1 peptide (184–214) containing only the FDFEK motif binds to CGH-1_248–420_ with a *K*_D_ of ~ 43 μM by ITC assay in the same buffer conditions^17^. The binding affinity of EDC-3 FDF-FEK motif is more 126 times than that of the corresponding region of CAR-1 (0.34 μM (this work) vs 43 μM^17^).

To understand the molecular basis by which cause so big differences in the binding affinity for EDC-3 and CAR-1, both containing the FDF binding motif that is anchored to CGH-1 hydrophobic pocket, we analyzed the binding mode between EDC-3 and CAR-1 when they are bound to CGH-1, respectively. From the comparison of structural models, we found that both EDC-3 and CAR-1 utilize the FDF motif to bind to the hydrophobic pocket of the CGH-1 RecA2 domain (Patch 1; Fig. 3A–D). For the recognition of EDC-3 FDF motif, CGH-1 residues His_269_, Cys_270_, Leu_271_, Asn_272_, Thr_273_, and Leu_274_ may involve (Fig. 2A). For the recognition of CAR-1 FDF motif, CGH-1 residues Ala_260_, Val_262_, His_269_, Cys_270_ and Leu_274_ might be involved^17^. The patterns of both Patch 1 are similar though the details are not exactly same (Fig. 3A,B). In other words, Patch 1 should not be the basic reason that make the big differences in the binding affinity of EDC-3 and CAR-1 when each of them is bound to the C-terminal RecA-like domain (RecA2) of CGH-1. There are some other reasons those might be critical to affect the binding affinity between EDC-3 and CAR-1when each is bound to CGH-1 RecA2.

We also investigated the differences in the binding modes between EDC-3 and CAR-1 for CGH-1, which is mainly reflected in the Patch 2 of the EDC-3/CGH-1 complex and Patch 3 in the CAR-1/CGH-1 complex (Fig. 3A–D). In EDC-3, the two CGH-1 binding sites FDF and FEK, which are respectively involved in Patches 1 and 2, are coupled by the helix H1 (Fig. 3A,C,E). In contrast to EDC-3, the FEK motif immediately follows the FDF motif, and they share the common phenylalanine Phe_192_ in CAR-1 (Fig. 3E). In CAR-1, only the FDF motif is involved in the interaction with CGH-1 (Patch 1), the Glu_193_ and Lys_194_ of CAR-1, in which the sidechains are opposite to CGH-1, are not able to interact with CGH-1 (Fig. 3D,E). Instead, the ETFG motif (residues 265–268) in the αC helix of CAR-1 docks to CGH-1 and forms the second binding site (Patch 3^17^). Taken all into together, we uncover the similarities and differences in the binding modes between EDC-3 and CAR-1 for CGH-1.

Coincidentally, the structural basis for the interactions of the P body components EDC3 and Tral (CAR-1 in *C. elegans*) with the DEAD-box RNA helicase Me31B (CGH-1 in *C. elegans*) are mutually exclusive in *D. melanogaster*, has been elucidated by structural biology combined with mutational and competition studies^25^.

### Similar binding mode between CAR-1/CGH-1 and *Sc*Pat1/*Sc*Dhh1p

Here, we have also discussed the binding modes in the complexes of CAR-1/CGH-1 and *Sc*Pat1/*Sc*Dhh1p. Intriguingly, by structural comparison of *Sc*Pat1/*Sc*DDh1p and CAR-1/CGH-1, we found that *Sc*Pat1 show the similar binding mode to CAR-1 (Supplementary Fig. S3C), the big difference is that the (E)TFG motif is located at the N-terminus of (D)FDF motif in the amino acid sequence of *Sc*Pat1, however, in the amino acid sequence of CAR-1, the (E)TFG motif is located at the C-terminus of αC helix (Patch 3).

*Ce*PATR-1 is a homologue of *Sc*Pat1 (Supplementary Fig. S3A and B). Though the (E/D)TFG motif is highly conserved, the (D)FDF motif is highly varied in the species from *C. elegans* to human (Supplementary Fig. S3A). In *C. elegans*, the corresponding residues are NAEL in *Ce*PATR-1 protein. On one side, in the CGH-1/*Ce*PATR-1 complex, the ETFG motif of *Ce*PATR-1 may also dock to CGH-1 to form Patch 3. The molecular interaction might be mediated by the hydrophobic interaction between Phe_355_ in the FDF motif of CGH-1 and PATR-1 TFG motif. This hypothesis was confirmed in our ITC assays where shows that the F355A mutation in CGH-1 almost abolished the binding of PATR-1_30–67_ peptide to CGH-1_248–420_ mutant (Fig. 5A). On the other side, CGH-1 residues His_269_, Cys_270_, Thr_273_ and Leu_274,_ which has been confirmed important for the recognition of EDC-3, are not involved in the binding of PATR-1 peptide to CGH-1 because of the absence of a typical FDF motif in PATR-1. This hypothesis was further confirmed by our ITC assays where shows the 4A mutant has the same binding affinity as the wild-type CGH-1 (Fig. 5B, Supplementary Fig. S4). However, a small hydrophobic patch including Phe_261_, Val_262_ and Tyr_386_ in CGH-1 may be involved in binding of CGH-1 to PATR-1 (this work), and these three residues also interact with CAR-1^17^. Whether PATR-1 NAEL motif can recognize this small hydrophobic patch need be further studied in our future work.

In summary, EDC-3 mainly employed its short linear motifs, including FDF motif and FEK motif, to interact with CGH-1 RecA2 domain. The results of sequence and structural alignments indicated that the recognition mechanism is conserved. Furthermore, we uncovered the similarity and differences in binding of EDC-3, CAR-1, or PATR-1 to CGH-1.

## Supplementary Information

Supplementary Information.
