# Supplementary material for: Insight into the interaction between the RNA helicase CGH-1 and EDC-3 and its implications
Source: Sci Rep. 2021 Oct 13;11:20359. doi: 10.1038/s41598-021-99919-0 (PMC8514580; doi:10.1038/s41598-021-99919-0)
Supplement: Supplementary file 1 — Supplementary Information. [file 41598_2021_99919_MOESM1_ESM.pdf]

Supplementary Information

Insight into the interaction between the RNA helicase CGH-1 and EDC-3 and its implications

Yong Zhang[1,2], Ke Wang[1,2], Kanglong Yang[1,2], Yunyu Shi[1,2], Jingjun Hong[1,2*]

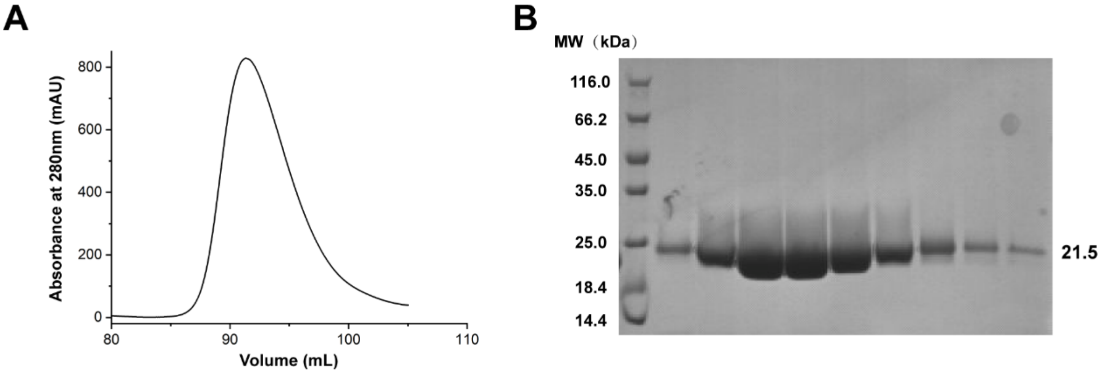

Figure S1. Protein purification. (A) Size exclusion chromatography (SEC) using a HiLoad 16/60 Superdex 75 column (GE Healthcare) for CGH-$1_{248\text{-}420}$. (B) The SDS-PAGE analysis of fractions of CGH-$1_{248\text{-}420}$ from (A).

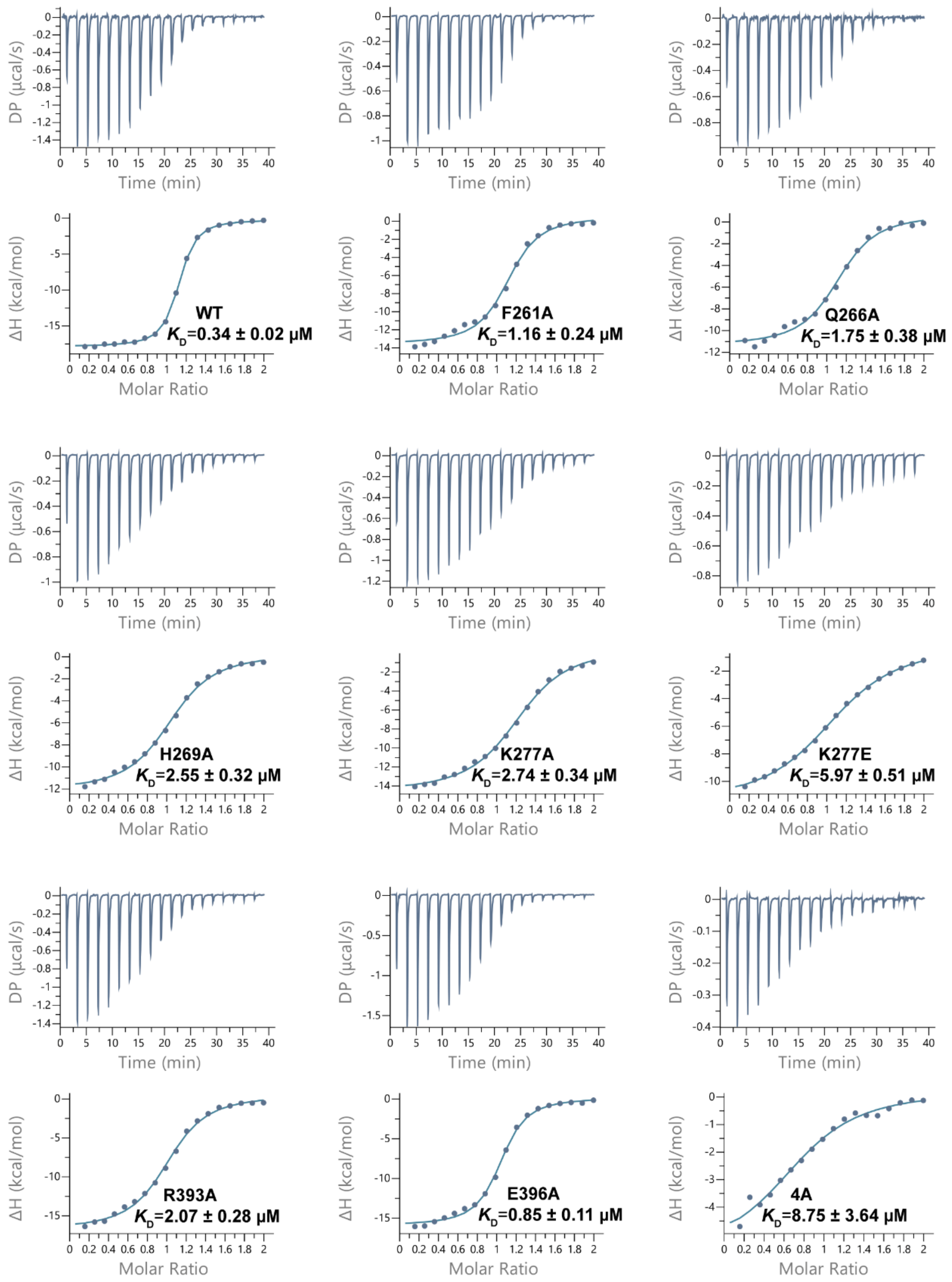

Figure S2. The isothermal titration calorimetry and fitting curves of CGH-1$_{248-420}$ (WT or mutants) and EDC-3 FDF-FEK peptide.

A

```
Ce_PATR1    30 SDE....DHNIFDDEFDAANDETFGGGLDNIGENAELENYAT 67
Sc_PAT1     25 GYG....EHEL..EENDYLNDETFGDNVQ.VGTDFDFGNPHS 59
Hs_PATL1    21 GLG....E.ED..EEIDQFNDDTFGSGA....VDDDWQEAHE 51
Xl_PATL1     7 GDGFPDLE.ED..EDIDQFNDDTFGAGA....VDDDWREEHE 41
Dm_PATR1    20 GRI......VE..AEYDALNDETFGSAI.....NGDWEEAHE 48
```

B

```
Ce_PATR1    30 SDE....DHNIFDDEFDAANDETFGGGLDNIGENAELENYAT 67
Sc_PAT1     25 GYG....EHEL..EENDYLNDETFGDNVQ.VGTDFDFGNPHS 59
Ce_CAR1    184                             LKFESDFDFEKANE
               KFQEVLVDNLEKLNIEDKAEPEVEEKKDAAFYDKKTSFFDNI
               SCESLEKAEGKTGRPDWKKERETNQETFG              268
```

C

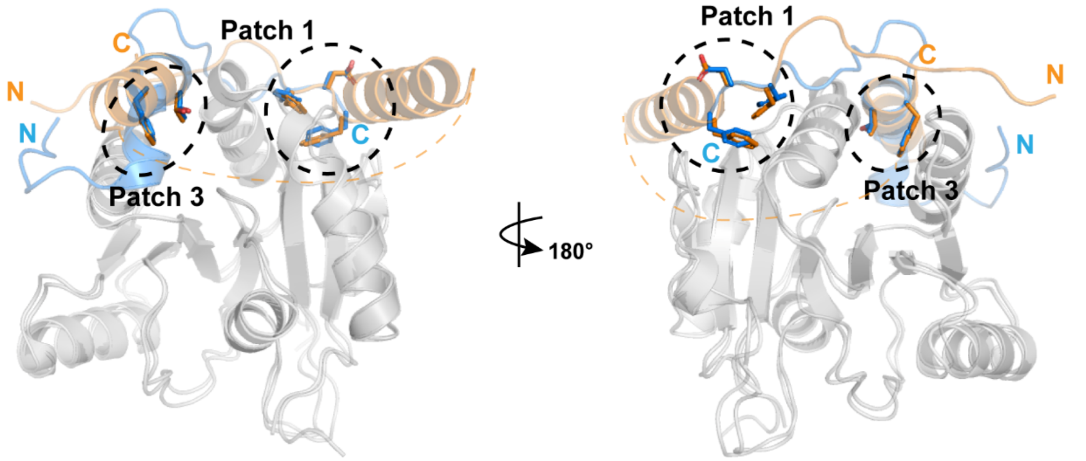

Figure S3. Sequence alignment and structural comparisons.

(A) Multiple sequence alignment of *S. cerevisiae* Pat1 (residues 25-59) and its homologs in *C. elegans* (PATR-1), in *D. melanogaster* (PATR1), in *X. laevis* (PATL1), and in *H. sapiens* (PATL1). The conserved TFG motif and varied region which is corresponding to the DFDF motif of *Sc*Pat1 were highlighted in color magenta, respectively.

(B) The comparison of the amino acid sequence of *S. cerevisiae* Pat1 (residues 25-59), *C. elegans* PATR-1 (residues 30-67) and CAR-1 (residues 184-268). The two regions that are corresponding to the TFG and DFDF motifs were highlighted in magenta, respectively.

(C) Structural comparison between *C. elegans* CAR-1/CGH-1 complex and *S. cerevisiae* Pat1/Dhh1p complex. The similar modes of interactions were shown in this panel. CAR-1 is colored in orange, Pat1 in marine blue, and both RecA2 domains of CGH-1 and Dhh1p are in light grey. The PDB code 4Brw for the crystal structure of *S. cerevisiae* Pat1/Dhh1p complex. The Mode for *C. elegans* CAR-1/CGH-1 complex is reported in our most recent paper (Zhang *et al*., 2021).

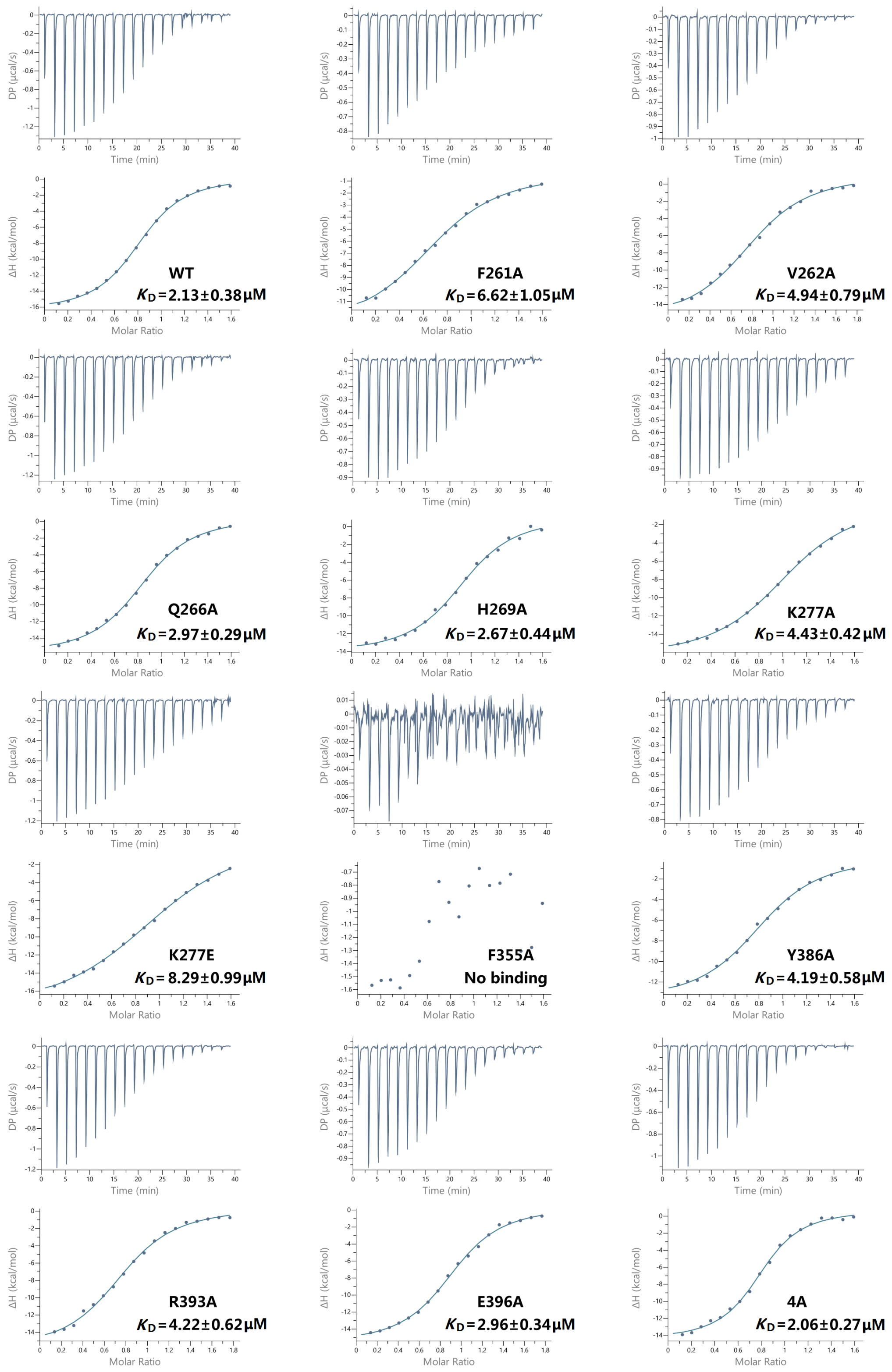

Figure S4. The isothermal titration calorimetry and fitting curves of CGH-1$_{248-420}$ (WT or mutants) and PATR-1$_{30-67}$ peptide. The working concentration for CGH-1$_{248-420}$ protein is 45 μM (V262A,

R393A, and E396A) or 50 μM (WT, or other mutants), and peptide concentration is 400 μM, respectively.

Table S1. Primers used for transgenes construction and sgRNA-mediated gene editing

3xFLAG-R	CTTCTTCACCCTTTGAGACCATcttgtcatcgtcatccttgtaatc
M+3xFLAG-F	atggactacaaagaccatgac
M-F1	ATGGTCTCAAAGGGTGAAGAAG
M-R1	AGCTCCACCTCCACCTCCCT
ori-amp F	tgtgaaattgttatccgctgg
ori-amp R	caCACGTGctggcgttacc
CGH-1_LA_F1	gttgggtaacgccagCACGTGtgtcttggatctgcttatttc
CGH-1_LA_R1	AAAGTTCTTCTCCTTTACTCATtttccgatgtcgtagtagg
CGH-1_RA_F1	CAAAGGAGGTGGAGGTGGAGCTATGAGTGGAGCGGAGCAACAAC
CGH-1_RA_R1	ccagcggataacaatttcacaCTGCTGGTCAGCGACATACAG
EDC-3_LA_F1	gttgggtaacgccagCACGTGtgctgaaacacagacagcgg
EDC-3_LA_R1	gtcatggtctttgtagtccatttttaatttgaaatcagaag
EDC-3-RA_F1	AGGGAGGTGGAGGTGGAGCTATGGATGACAAACTCATTGGAAG
EDC-3_RA_R1	ccagcggataacaatttcacaGAAGATGCGATCGGCGACGATTG
G-F1	ATGAGTAAAGGAGAAGAACTTTTC
GFP-R1	AGCTCCACCTCCACCTCCTTTG
CGH-1-KI-sg1-F1	CAGATCGTTCCTGCCAACAAGTTTAAGAGCTATGCTGGAAAC
CGH-1-KI-sg1-R1	TTGTTGGCAGGAACGATCTGCAAACATTTAGATTTGCAATTC
CGH-1-KI-sg2-F1	GacatcggaaaATGAGTGGAGGTTTAAGAGCTATGCTGGAAAC
CGH-1-KI-sg2-R1	CTCCACTCATtttccgatgtCAAACATTTAGATTTGCAATTC
CGH-1-KI-sg3-F1	GctacgacatcggaaaATGAGGTTTAAGAGCTATGCTGGAAAC
CGH-1-KI-sg3-R1	CTCATtttccgatgtcgtagCAAACATTTAGATTTGCAATTC
EDC-3-KI-sg4-F1	GtaatttgaaatcagaagaatGTTTAAGAGCTATGCTGGAAAC
EDC-3-KI-sg4-R1	attcttctgatttcaaattaCAAACATTTAGATTTGCAATTC
EDC-3-KI-sg5-F1	GaaaATGGATGACAAACTCAtGTTTAAGAGCTATGCTGGAAAC
EDC-3-KI-sg5-R1	ATGAGTTTGTCATCCATtttCAAACATTTAGATTTGCAATTC
EDC-3-KI-sg6-F1	GGACATTTCCATCTTTAGTTTGTTTAAGAGCTATGCTGGAAAC
EDC-3-KI-sg6-R1	AAACTAAAGATGGAAATGTCCAAACATTTAGATTTGCAATTC
